# EUS-guided gastroenterostomy using a novel through-the-scope exchangeable dual-balloon enteroclysis catheter: a potentially secure and scalable approach

**DOI:** 10.1016/j.vgie.2023.07.014

**Published:** 2023-09-12

**Authors:** Yen-I Chen, Charles Menard, Mouen Khashab, Gary May, Corey Miller, Nauzer Forbes, Sheryl White, Ali Bessissow

**Affiliations:** 1 Division of Gastroenterology and Hepatology, McGill University Health Centre, McGill University, Montreal, Quebec, Canada; 2 Division of Gastroenterology, University of Sherbrooke, Sherbrooke, Quebec, Canada; 3 Division of Gastroenterology and Hepatology, Johns Hopkins Hospital, Baltimore, Maryland; 4 Division of Gastroenterology, St-Michael’s Hospital, University of Toronto, Ontario, Canada; 5 Division of Gastroenterology, Jewish General Hospital, McGill University, Montreal, Quebec, Canada; 6 Division of Gastroenterology, University of Calgary, Calgary, Alberta, Canada; 7 Division of Gastroenterology and Hepatology, McGill University Health Centre, McGill University, Montreal, Quebec, Canada; 8 Department of Radiology, McGill University Health Centre, McGill University, Montreal, Quebec, Canada

## Abstract

Video 1 Clinical case of dual-balloon through-the-scope exchangeable enteroclysis catheter-assisted EUS-guided gastroenterostomy.

Clinical case of dual-balloon through-the-scope exchangeable enteroclysis catheter-assisted EUS-guided gastroenterostomy.

Despite accumulating evidence of efficacy in the management of malignant gastric outlet obstruction (MGOO),^1^, ^2^, ^3^, ^4^ EUS-guided gastroenterostomy (EUS-GE) has yet to gain widespread adoption. The lack of a scalable technique and the fear of stent misdeployment has hampered its dissemination. Indeed, even in expert hands, stent misdeployments with EUS-GE have been shown to be as high as 6% to 27%.^1^^,^^2^^,^^5^, ^6^, ^7^ Procedure aids such as the specialized double occlusive balloon enteric tube have been developed. This technique, coined EUS-guided double-balloon-occluded gastrojejunostomy bypass, allows the isolation of the targeted small bowel with 2 balloons and optimal distention of the isolated segment with infusion of fluid using a separate port in the specialized tube.^8^^,^^9^ Despite greatly facilitating EUS-guided stent insertion, this catheter is limited by its cumbersome insertion, as it cannot be advanced or exchanged through the endoscope.

We describe a proprietary dual-balloon through-the-scope exchangeable enteroclysis catheter to facilitate EUS-GE (DUBX, Naja; Chess Medical, Montreal, Quebec, Canada). The DUBX has 2 occlusive balloons to allow for enhanced enteroclysis of an isolated small-bowel segment. The balloons are 12 cm apart and can be inflated up to 50 mm in diameter using air (Fig. 1). It is inserted through an endoscope with a 3.7-mm working channel and can be exchanged through this channel, allowing the catheter to remain at the intended location during endoscope removal. The working hub is attachable and detachable with a plurality of infusion lumens for independent inflation of the balloons, infusion of fluid between the balloons, and accommodation of a guidewire (Fig. 2).

**Figure 1:**
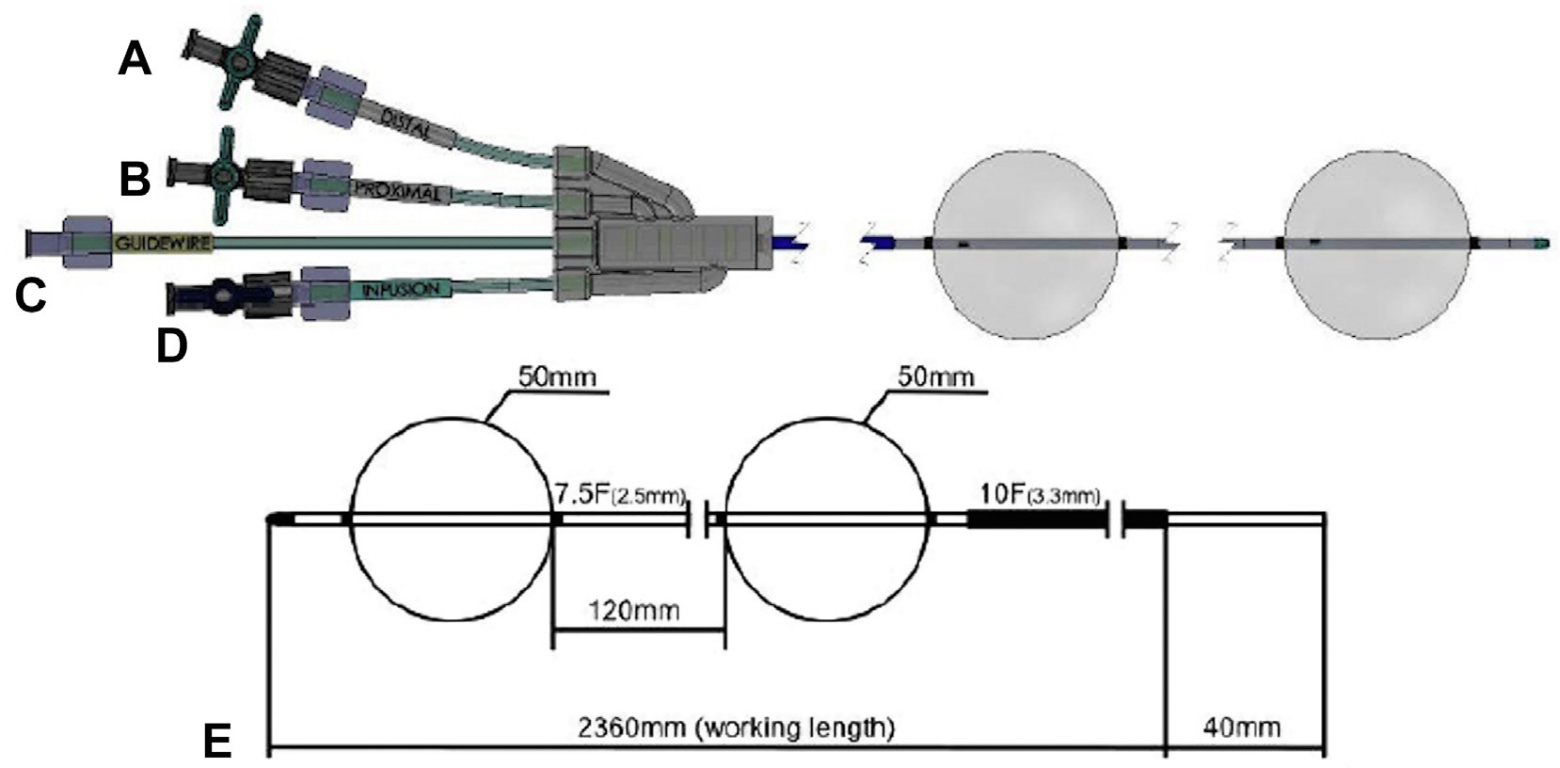
Dual-balloon exchangeable catheter. **A,** Distal balloon infusion port. **B**, Proximal balloon infusion port. **C,** Guidewire port. **D**, Fluid infusion port for small-bowel distension. **E**, Catheter and balloon dimensions.

**Figure 2:**
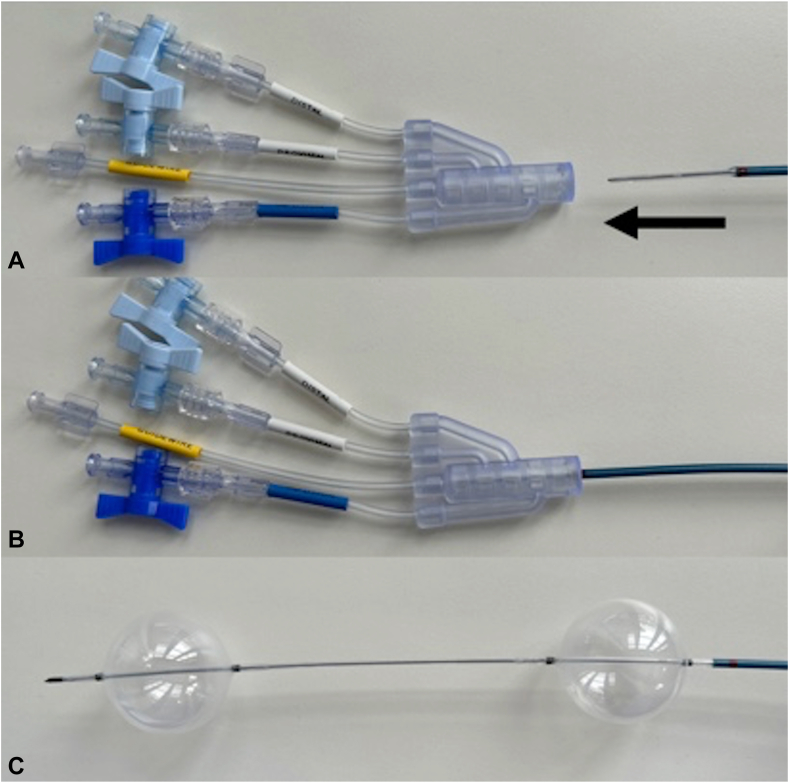
**A**, Working hub detached from proximal tip of the enteroclysis catheter. **B**, Working hub attached to proximal tip of the enteroclysis catheter. **C**, Inflation of both the distal and proximal balloon to 50 mm with enteroclysis infusion skives located between the 2 balloons.

The first clinical case of DUBX was performed under the approval of Health Canada’s Special Access Program. A 65-year-old man with advanced, unresectable gastric cancer involving the gastric antrum presented with recurrent MGOO post–enteral stent insertion. Following informed consent, the procedure was performed with the patient in the supine position under general anesthesia (Video 1, available online at www.videogie.org). A therapeutic gastroscope (GIF-1TH190; Olympus, Central Valley, Penn, USA) was advanced to the antrum. A high-grade stricture was noted at the antrum with complete tumor tissue stent ingrowth. A 0.035-inch wire was advanced across this obstruction into the jejunum. The DUBX was then loaded into the wire and advanced through the endoscope to the ligament of Treitz under fluoroscopic guidance. The endoscope was then removed via exchange over the DUBX. The attachable working hub was then connected to the catheter. The distal balloon was inflated to 40 mm using air followed by inflation of the proximal balloon to the same volume. Infusion of 150 mL of saline mixed with methylene blue and contrast was then performed through the infusion port of the working hub into the occluded segment of the small bowel (Fig. 3). A therapeutic linear echoendoscope (GF-UCT180, Olympus) was then inserted with excellent views of the distended occluded small-bowel segment. A 20-mm lumen-apposing metal stent was then inserted freehand with cautery assistance and deployed without adverse event. During the cautery-assisted stent insertion, the small-bowel remained tightly juxtaposed to the stomach without indentation or collapse. The patient was discharged from the hospital 5 days later tolerating a low-residue diet.

**Figure 3:**
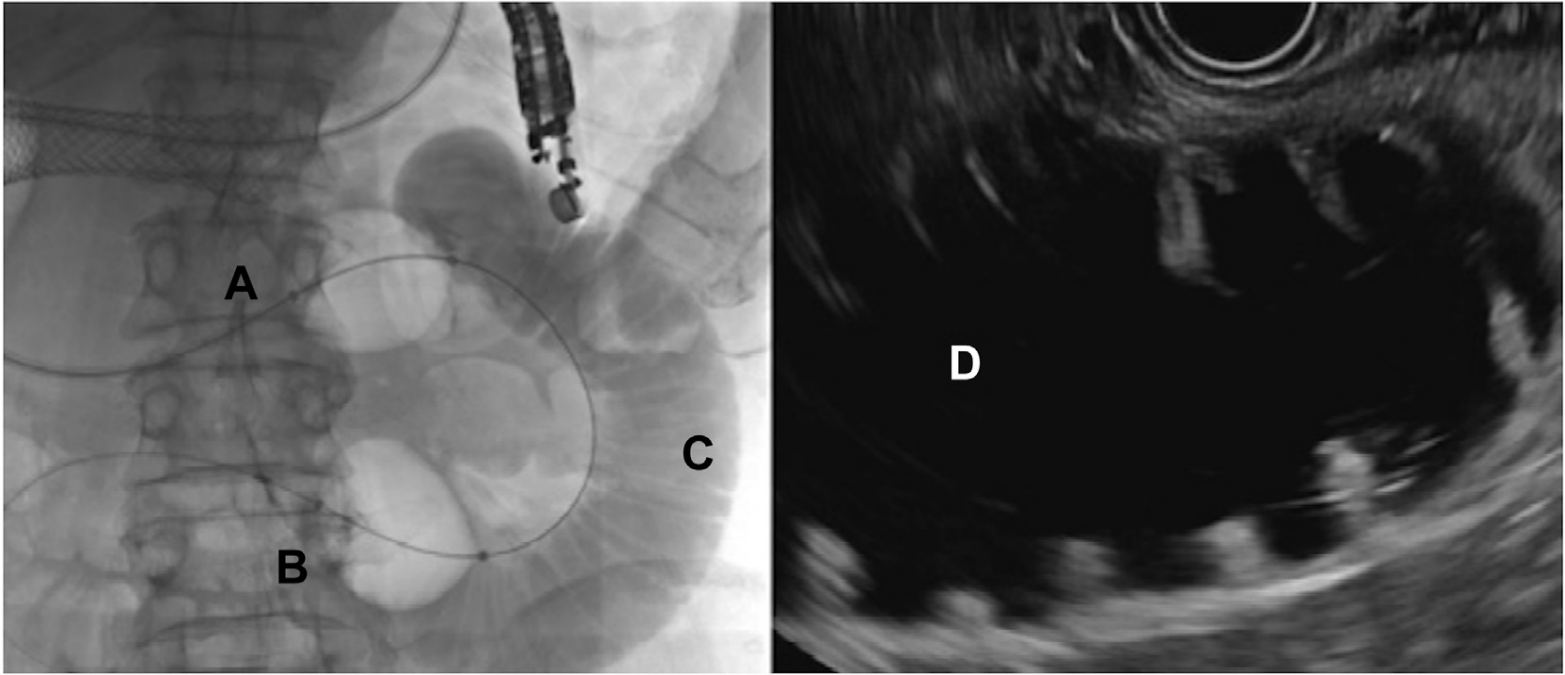
Complete interballoon occlusion and small-bowel space fluid/contrast distention with no evidence of leakage across balloons. **A,** Proximal balloon inflated with 40 mL of air. **B,** Distal balloon inflated with air. **C,** One hundred fifty milliliters of contrast mixed with saline infused between the balloons in the small bowel. **D,** EUS view of the targeted enteroclysis.

EUS-GE is a promising modality whose widespread use is currently limited by the lack of a scalable, standardized technique. The DUBX is a simple and likely scalable solution. This first clinical case demonstrates its ability to achieve targeted enteroclysis with optimal bowel distension while being deliverable through the endoscope. This novel catheter has the potential to greatly enhance the safety and ease of EUS-GE with the goal of enabling dissemination of this technique beyond the walls of expert centers. Clinical studies are needed.

## Disclosure

Dr Chen is a consultant for and has received funding from Boston Scientific, has received research funding and is a cofounder and shareholder of Chess Medical, and has received funding from the Fonds de Recherche du Québec - Santé (FRQS) and the Canadian Institute of Health Research. Dr Khashab is a consultant for Boston Scientific and an advisor to Chess Medical. Dr Forbes is a consultant for Boston Scientific and Pentax. Dr May is a consultant for Boston Scientific, Olympus Medical, and Fujifilm Endoscopy. Dr Bessissow is a consultant for Boston Scientific and is a cofounder and shareholder of Chess Medical. The other authors did not disclose any financial relationships.

## Supplementary data

Video 1 Clinical case of dual-balloon through-the-scope exchangeable enteroclysis catheter-assisted EUS-guided gastroenterostomy.
